# Determining the Magnetic Properties of 1 kg Mass Standards

**DOI:** 10.6028/jres.100.015

**Published:** 1995

**Authors:** Richard S. Davis

**Affiliations:** Bureau International des Poids et Mesures, Pavillon de Breteuil, F-92312 Sèvres Cedex, France

**Keywords:** magnetic permeability, magnetic susceptibility, mass metrology, mass standards, susceptometer

## Abstract

Magnetic interactions may lead to errors in precision mass metrology. An analytical description of such magnetic errors is presented in which the roles of both the volume magnetic susceptibility and permanent magnetization are discussed. The same formalism is then used to describe in detail the calibration and operation of a susceptometer developed at the Bureau International des Poids et Mesures (BIPM). The device has been optimized for the determination of the magnetic properties of 1 kg mass standards.

## 1. Introduction

Suppose that a mass standard having a small but finite magnetic susceptibility or permanent magnetization has been placed on one pan of a beam balance. The magnetic properties of the standard may lead to a potential energy term that also depends on the magnetic fields within the balance. If this magnetic potential energy changes as a function of rotation of the balance beam, then there will be an unwanted torque on the beam. One may also note that rotation of the beam through small angles is equivalent to motion of the mass standard in the vertical direction. From this qualitative argument, it is clear that a magnetic potential due to the mass standard and any balance components fixed with respect to the frame of reference of the standard can pose no problems to weighing. Problems, when they occur, are due to the magnetic properties of the mass standard and local magnetic fields that are in a frame of reference (usually that of the laboratory) that moves relative to the standard on the balance pan. In order to quantify such problems it is necessary to characterize both the magnetic properties of the mass standard and the magnetic environment of the laboratory. This paper will concentrate on the former task.

In a previous publication [1] we showed how the volume magnetic susceptibility *χ* of nonmagnetic materials could be measured by simple modification of a commercial microbalance. Tests carried out at the Bureau International des Poids et Mesures (BIPM) verified that a reasonably uncomplicated theory of operation is adequate to achieve results that have a relative combined standard uncertainty of a few percent over the range of susceptibilities encountered in mass standards of high quality. (Throughout this report uncertainties are expressed according to guidelines set forth by the International Organization for Standardization [2].)

In this report, we first discuss a model for errors in mass metrology due to magnetic effects. These are shown to depend mainly on the susceptibility and the permanent magnetization of the mass standard. The latter is zero for nonmagnetic materials but some materials used in the manufacture of good mass standards are, in fact, weakly magnetic and thus may become magnetized.

Susceptibility can be measured quantitatively using the BIPM apparatus and permanent magnetization can be detected. These measurements are discussed in detail, including calibration of the instrument and estimation of measurement uncertainty. Of equal importance to making good measurements is interpreting their significance. We therefore begin with a basic review of the most probable magnetic problems in precision mass measurement and conclude with several practical examples.

## 2. Model for Magnetic Errors in Mass-Metrology

By magnetic error, we mean an unsuspected vertical force *F* that is magnetic in origin. Such a force will be misinterpreted as a mass *F*/*g*, where *g* is the local acceleration of gravity.

We may assume that high-quality mass standards are artifacts with an isotropic volume magnetic susceptibility *χ* whose magnitude is much less than one. In addition, artifact mass standards should have little or, ideally, no permanent magnetization. Thus the unwanted magnetic force will, to a good approximation, be given by

$$F=-\frac{\mu_{0}}{2}\frac{\partial }{\partial z}\int \chi^{\prime }H\cdot HdV-\mu_{0}\frac{\partial }{\partial z}\int M\cdot HdV,$$ (1)

where *χ*′ is the effective volume magnetic susceptibility of the standard, ***M*** is its permanent magnetization (defined as the magnetic moment per unit volume in zero field), ***H*** is the local magnetic field strength and the *z*-axis is parallel to *g*. In general, all these terms may depend on position but we assume that *χ*′ is a scalar. The parameter *μ*_0_ is the vacuum permeability, identically equal to 4*π* × 10^−7^ N·A^−2^. The integrals are taken over the volume of the artifact. The effective susceptibility *χ*′ is defined as *χ*− *χ*_A_, where *χ*_A_ is the volume susceptibility of air (+3.6 × 10^−7^). Because of its relatively small magnitude, *χ*_A_ can be neglected in most of the examples given below. The symbols, quantities and nomenclature used in this report are those set forth by the International Union of Pure and Applied Physics [3].

We have assumed in Eq. (1) that ***H*** is the field before the sample is introduced, an approximation good to first order in the susceptibility. We have also assumed that the alloy is linear, i.e., its susceptibility is independent of applied magnetic field for strengths less than, say, 4 kA·m^−1^. Finally, we have assumed that the effect of a permanent magnetization ***M*** can simply be added as a term separate from the induced magnetization *χ*′***H***. The validity of this approach will be demonstrated by examples given below. Note that, for a mass standard to be magnetized, it must have been exposed to field strengths greater than the linear threshold.

We can further simplify Eq. (1) by the additional assumption, usually warranted, that the linear and isotropic susceptibility is also homogeneous throughout the artifact. Finally, we make an *un*warranted assumption that ***M*** is a constant in magnitude and direction throughout the artifact and is independent of ***H*** at low field strength. It would be difficult to proceed without the last assumption but the reader should keep in mind that its importance in what follows is largely heuristic. This allows us to write a simplified force equation:

$$\begin{array}{c} F=-\frac{\mu_{0}}{2}\chi^{\prime }\frac{\partial }{\partial z}\int H\cdot HdV-\mu_{0}M_{z}\frac{\partial }{\partial z}\int H_{z}dV\\ -\mu_{0}M_{x}\frac{\partial }{\partial z}\int H_{x}dV-\mu_{0}M_{y}\frac{\partial }{\partial z}\int H_{y}dV. \end{array}$$ (2)

It should be noted that *M_z_, M_x_* and *M_y_* are the vertical and horizontal components of ***M*** and thus change with the orientation of the artifact. Without knowledge of the magnetic fields and gradients within the balance our model can take us no further except to imply the following: (i) There can be no magnetic errors if there are no field *gradients* in the balance, and (ii) Due to the symmetry of certain shapes (e.g., cylinders) some artifacts can be turned upside-down without changing the force contribution from the susceptibility. The sign of *M_z_* is, by contrast, reversed for these shapes.

Two serious attempts have been made to apply Eq. (2) to problems of mass metrology. Gould [4] presented several special cases to illustrate his contention that the worst problems are likely to occur when the mass standard and a part of the balance case (including the mass transporter in automated balances) located just below the pan are both accidentally magnetized along the vertical axis. This argument implies that the second integral in Eq. (2) is the most important contribution, the fields and gradients arising primarily from the magnetized parts of the balance.

Using this reasoning as a guide for the selection of suitable stainless steel alloys, Gould concluded that an alloy used for mass standards should be chosen both for its low susceptibility and for its resistance to permanent magnetization upon exposure to high fields. He found that for stainless steels, these two desirable properties are well correlated. That is, the alloy with the lowest susceptibility was also the most difficult to magnetize. The recommended alloy was found to have *χ* ≈ 0.003 when measured in a uniform field strength of 16 kA·m^−1^. The permanent magnetization was less than 1 A·m^−1^ after exposure to a “suitably large” uniform field (we have converted values given in Ref. [4] from CGS-EMU to SI [3]).

Kochsiek [5] approached the problem in a different way. He dealt with Eq. (2) by arguing that, once demagnetized, the normal use of mass standards should not subject them to fields great enough to remagnetize them. A strong recommendation was made against selecting inferior stainless steel alloys, known to be easily magnetized. He then chose a special case in which the integrals in Eq. (2) may be solved trivially: the magnetic field within the balance chamber is vertical with the form

$$H_{z}=h_{0}+h_{1}z,$$ (3)

where *h*_0_ and *h*_1_ are constants and the origin of the coordinate system is at the geometric center of the mass standard. As an example, *h*_0_ was chosen to be 100 A·m^−1^ and *h*_1_ to be 5000 A·m^−2^. (As a point of reference, the vertical component of the Earth’s magnetic field strength is about 40 A·m^−1^ at the latitude of Paris.) Based on this somewhat arbitrary choice of “worst-case” parameters, it was then possible to argue that secondary 1 kg mass standards used by national laboratories should have a volume susceptibility below 0.003.

It is instructive to note that this recommendation also allows us to calculate an upper limit for *M_z_* in Eq. (2): |*M_z_*| < 0.3 A·m^−1^. The limit is derived from Eqs. (2) and (3) with the condition that the second integral in Eq. (2) be smaller than the first when *χ*′ = 0.003. In general for this model, *M_z_* is negligible when |*M_z_* | < | *χ*′*h*_0_|.

Thus we see that Refs. [4] and [5], while focusing on different aspects of the problem, arrive at nearly the same guidelines for selecting stainless steel alloys suitable for the highest quality of secondary mass standards. Nevertheless, it should be emphasized that Eq. (2) cannot be solved without detailed knowledge of the fields within the balance. The Organisation Internationale de Métrologie Légale (OIML) has recently recommended that Class E_1_ and E_2_ mass standards have volume susceptibilities less than 0.01 and 0.03 respectively [6].

## 3. Susceptometer Developed at the BIPM

**Fig. 1:**
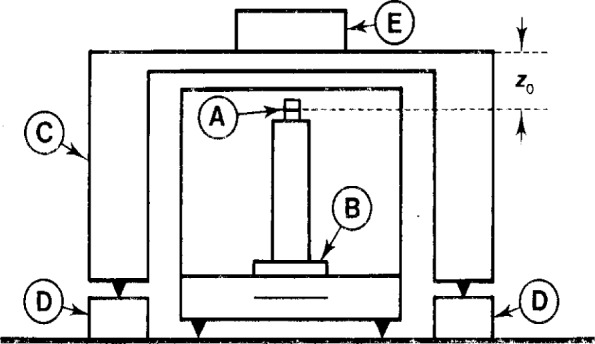
Schematic view of the apparatus. A small rare-earth magnet (A) is placed on a supporting column which rests on the balance pan (B). A nonmagnetic bridge (C), the height of which may be incremented with gauge blocks (D), straddles the balance. The sample (E) is placed on the bridge, directly above the magnet.

The BIPM susceptometer subjects a mass standard to relatively small, calculable fields, measures the resulting vertical force and then makes use of Eq. (2) to find the susceptibility and the parameter *M_z_*. The principles of construction are given in Ref. [1] but we show the apparatus schematically in Fig. 1 and give design details in Appendix A. The magnet that we use is cylindrical with height *L* and diameter both equal to 5 mm.

The balance reads in units of mass, the reading becoming more negative when a paramagnetic sample is introduced. Thus, the following relation is used:

+1μNcorresponds to a blance reading of−101.9μg,

where the acceleration of gravity *g* has been taken to be 9.81 m·s^−2^.

### 3.1 Response to Large Samples

Some insight can be gained by considering a sample in the form of a semi-infinite slab and approximating the magnet as being uniformly magnetized along its axis. If the magnet were a sphere, the first integral in Eq. (2) would have a particularly simple solution [1]:

$$F=\frac{3\mu_{0}\chi^{\prime }m^{2}}{64\pi Z_{0}^{4}}\equiv \chi^{\prime }F_{\max},$$ (4)

where *m* is the moment of the magnet (Sec. 4) and *Z*_0_ is the distance between the center of the magnet and the sample, as shown in Fig. 1. The remaining integrals vanish in this case. A more general result, correct to all orders of *χ*, could have been derived using the method of images.

**Fig. 2:**
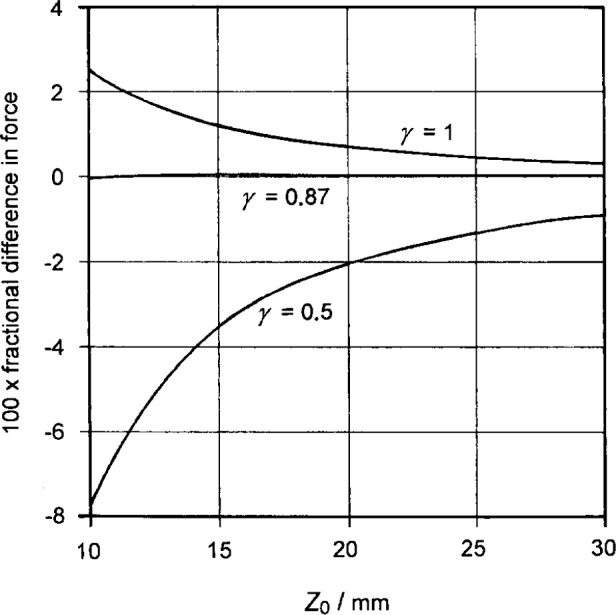
Expected behavior of three cylindrical magnets of different aspect ratio (height/diameter) *g* relative to a spherical magnet. All four magnets have the same volume (98 mm^3^) and uniform magnetization. The case considered is the attractive force between each magnet and a semi-infinite slab of small susceptibility. The spherical magnet is taken as the reference and is thus represented by the abscissa.

The method of images may also be applied to the case of the cylindrical magnet actually used, modeling the magnet as a cylindrical sheet of uniform current. The solution involves elliptic functions that can be calculated numerically. Only the first integral of Eq. (2) is not zero, just as in the case of a spherical magnet. In Fig. 2, force calculations for cylindrical magnets of different aspect ratio *γ* (height/diameter) are compared with calculations for a spherical dipole, where all magnets have the same volume and the same value of uniform magnetization. The sample is again assumed to be a semi-infinite slab of small susceptibility. It is, of course, well known that cylindrical magnets cannot be uniformly magnetized but the approximation is a good one for the neodymiumiron-boron magnets which we use.

For large samples, a cylindrical magnet with *γ* = 1 thus produces almost the same force as a spherical magnet of equal moment. The difference is within 2.6 % for *Z*_0_ > 2*L* and within 1.2 % for *Z*_0_ > 3*L*. Even closer agreement would be expected if *γ* were 0.87 instead of 1. However, the usual shape of cylindrical rare-earth magnets has *γ* = 0.5, so it is convenient simply to combine two such magnets.

Using the property of superposition, we can immediately find the force on a slab of finite thickness *t*. If we define *Z*_1_ = *Z*_0_ + *t*, then

$$F=\chi^{\prime }F_{\max}\left[1-{\left(\frac{Z_{0}}{Z_{1}}\right)}^{4}\right]=\chi^{\prime }F_{\max}\left[1-Z_{\ln}^{-4}\right]$$

where the dimensionless quantity *Z*_1n_ is the length *Z*_1_ normalized to *Z*_0_.

More generally, we next consider how large a cylindrical sample of radius *c* and height *t* must be before it may be approximated as “semi-infinite.” To do this, we insert the dipole fields due to the magnet in the first integral of Eq. (2) and solve for the case of cylindrical samples of finite dimensions. The result of the calculation is given in Eqs. (5) and (6).

**Fig. 3:**
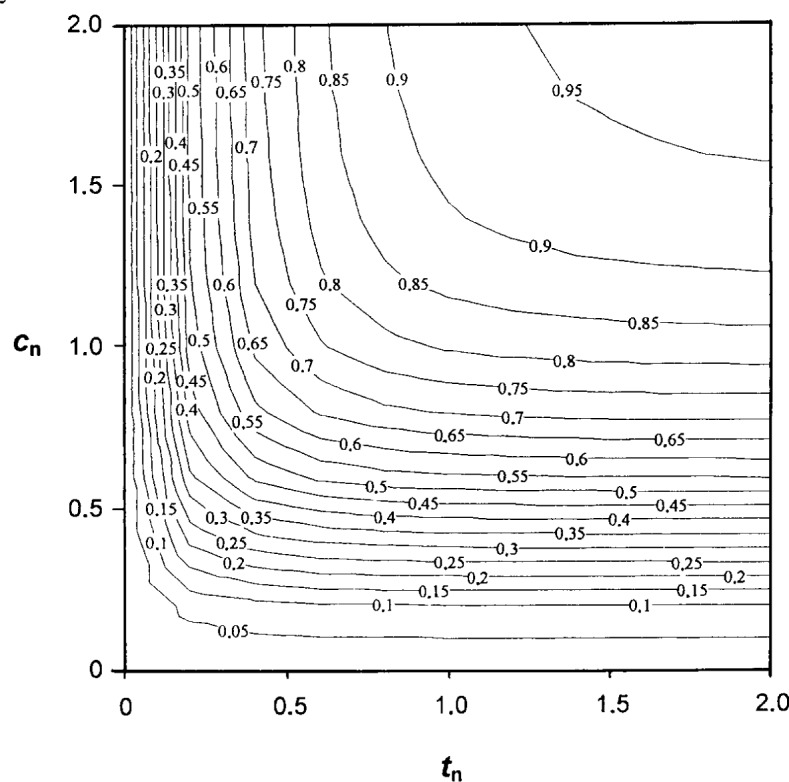
Contours of constant force between a dipole magnet and coaxial cylinders of varying radius *c*_n_ and thickness *t*_n_ but having the same, small susceptibility. Cylinder dimensions are normalized relative to *Z*_0_, the distance between the center of the magnet and the base of the cylinders. Force contours are normalized to the signal from a cylinder of infinite radius and height (i.e., a semi-infinite slab).

Figure 3 shows contours of equal force between the magnet and samples of differing dimensions. The force is normalized to *χ*′*F*_max_ and the cylinder dimensions are normalized to *Z*_0_. Only a finite volume of semi-infinite samples contributes strongly to the force integral. The important volume is of order $Z_{0}^{3}$ and thus changes as *Z*_0_ is varied experimentally.

### 3.2 General Equations

A sample placed on the susceptometer as shown in Fig. 1 will be subjected to fields from the cylindrical permanent magnet (***H***_mag_) and the Earth (***H***_E_)^1^. Making the dipole approximation for the cylindrical magnet and taking the Earth’s field to be uniform leads to the following force equation [1]

$$F=\chi^{\prime }F_{\max}I_{a}+\frac{\mu_{0}}{4\pi }(\chi^{\prime }H_{Ez}+M_{z})\frac{m}{Z_{0}}I_{b}\equiv F_{a}+F_{b},$$ (5a)

where

$$\begin{array}{l} I_{a}=-\frac{32\pi }{3m^{2}}\frac{\partial }{\partial Z_{0n}}\int \int_{V_{n}}\int H_{\text{mag}}\cdot H_{\text{mag}}\;dV=\\ -\frac{2}{3\pi }\frac{\partial }{\partial Z_{0n}}\int \int_{V_{n}}\int \frac{\rho^{2}+4z^{2}}{{\left(\rho^{2}+z^{2}\right)}^{4}}\rho d\rho d\theta dz, \end{array}$$ (5b)

$$\begin{array}{l} I_{b}=-\frac{4\pi }{m}\frac{\partial }{\partial Z_{0n}}\int \int_{V_{n}}\int {\left(H_{\text{mag}}\right)}_{z}dV=\\ -\frac{\partial }{\partial Z_{0n}}\int \int_{V_{n}}\int \frac{\rho^{2}-2z^{2}}{{\left(\rho^{2}+z^{2}\right)}^{5/2}}\rho d\rho d\theta dz \end{array}$$ (5c)

and *F*_max_ is defined in Eq. (4). We have assumed the samples to be centered symmetrically about the magnet axis so that only the vertical components of ***H***_E_ and ***M*** contribute to the force. The integrals are taken over the normalized volume of the sample. It is evident from Eq. (5a) that the induced magnetization *χ*′*H*_E_*_z_* has the same effect as a permanent magnetization *M_z_*. We show below how the two contributions may, in practice, be distinguished. The differentiated integrals of Eqs. (5b) and (5c) are shown in detail for cylindrical coordinates where the origin is, as always, at the center of the magnet.

In practice, an initial force measurement *F*_1_ is made with the north pole of the magnet pointing down and a second measurement *F*_2_ is made (at the same *Z*_0_) with the north pole pointing up. Then the first term in Eq. (5a) is given by *F*_a_ = (*F*_1_ + *F*_2_)/2 and the second term by *F*_b_ = (*F*_1_ − *F*_2_)/2. It was noted in Ref. [1] that *Z*_0_ changes slightly each time the magnet is repositioned. In practice, we have not found this to be a problem for nominal settings of about 15 mm or greater.

From a measurement of *F*_a_ using the unknown sample and a knowledge of *m*, *Z*_0_ and the sample dimensions, we can solve for *χ*′, using Eqs. (4), (5a) and (5b). Using this *χ*′, the measured value of *F*_b_, and a knowledge of *H*_E_*_z_*, we can then find *M_z_* from Eqs. (5a) and (5c). The uniform magnetization is usually negligible. The quantities *I*_a_ and *I*_b_ are readily written in closed form for cylindrical samples of radius *c* and height *t* coaxial with the magnet. This is because each differentiated triple integral reduces to a single integral in this case. We repeat the relations, given in Ref. [1], using normalized variables^2^:

$$I_{a}=1-Z_{\ln}^{-4}-\frac{1+c_{n}^{2}/3}{{\left(1+c_{n}^{2}\right)}^{3}}+Z_{\ln}^{-4}\frac{1+{\left(c_{n}/Z_{\ln}\right)}^{2}/3}{{\left[1+{\left(c_{n}/Z_{\ln}\right)}^{2}\right]}^{3}}$$ (6a)

and

$$I_{b}=2\pi \left[\frac{c_{n}^{2}}{{\left(1+c_{n}^{2}\right)}^{3/2}}-\frac{c_{n}^{2}/Z_{\ln}^{3}}{{\left[1+{\left(c_{n}/Z_{\ln}\right)}^{2}\right]}^{3/2}}\right],$$ (6b)

where *Z*_1n_ ≡ 1+ *t*_n_. Note that 0 ≤ *I*_a_ ≤ 1, the upper limit being the case of a semi-infinite slab and the lower limit a sample of vanishingly small volume. In contrast, 0 ≤ *I*_b_ ≤ 2.42, the lower limit being obtained for both a semi-infinite slab and a sample of vanishingly small volume. For shapes other than cylinders, solutions can be found by numerical integration and/or superposition of easily calculable shapes. Several examples are given below and in Appendices B and C.

It can be shown in general that

$$\frac{F_{a}}{F_{b}}=\frac{3\pi }{8}\frac{\chi^{\prime }H_{\max}}{\chi^{\prime }H_{\text{Ez}}+M_{z}}\frac{I_{a}}{I_{b}},$$

where

$$H_{\max}\equiv \frac{m}{2\pi Z_{0}^{3}},$$

the magnitude of the maximum field strength to which the sample is subjected by the magnet alone. This is the field at the base of the sample, directly above the magnet. An interesting special case, which is not unusual, is that of a cylinder for which *Z*_1n_^4^ >> 1 and (*Z*_1n_/*c*_n_)^2^ >> 1. Then *I*_b_ takes its maximum value at $c_{n}=\sqrt{2}$, in which case *I*_a_/*I*_b_ = 0.4. Thus we see that, for a given *Z*_0_, an apparatus realized with a much more sensitive balance and a much smaller magnet than we use would favor the measurement of *F*_b_ instead of *F*_a_.

At any given setting of *Z*_0_, the geometric terms in Eq. (5) are the same for samples having the same dimensions and orientation. Thus the relative susceptibilities of congruent samples are easily determined from ratios of balance readings. To determine an unknown susceptibility, one might even machine a material of known susceptibility to match the dimensions of the unknown object [7]. Unless a large number of identical samples is to be determined, or the field of the magnet cannot be approximated as a dipole, this approach is less convenient than finding sufficiently accurate solutions to the differentiated integrals of Eqs. (5b) and (5c).

The susceptibilities of a set of objects, each having the same shape and size (such as similar mass standards), may be conveniently determined by determining the susceptibility of one and then treating it as a standard to find the susceptibility of the others through ratios of the observed values of *F*_a_.

It now remains to show how the BIPM susceptometer may be calibrated and how we use it.

## 4. Calibration of Susceptometer

Calibration of the device is equivalent to determining *m* and *Z*_0_. Several methods may be used and they give equivalent results. The two unknown parameters may, for example, be found by measuring two standards of different, known susceptibility. Such standards are not readily available in a suitable range of susceptibilities, however [8]. A reasonably good estimate of *m* can be inferred from technical information supplied with the magnet. As shown below, the susceptometer may also be self-calibrated by fitting measured values of *F*_a_ as a function of increments in *Z*_0_, obtained using a sample of unknown susceptibility, to the equations developed above. All these methods should, of course, be consistent within their combined uncertainties.

### 4.1 Determination of *m* From Supplier’s Specifications

At distances *Z*_0_ that are large compared with the magnet dimensions, the axial magnetic field strength due to the magnet is given by *H*_max_, and may be positive or negative depending on the orientation of the magnet. If the magnet has a uniform axial magnetization *M*, then *m* = *MV* where *V* is the volume of the magnet. It is more usual to characterize a permanent magnet by its polarization *J* = *μ*_0_*M*, which has units of tesla.

**Fig. 4:**
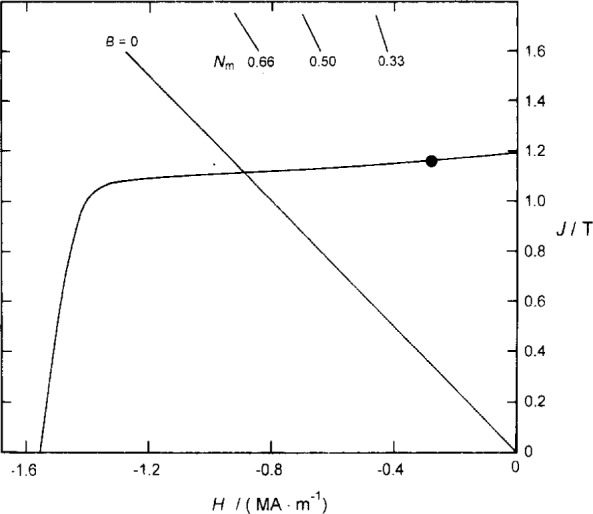
Depolarization curve supplied by the manufacturer of the magnet used in the susceptometer. The ordinate represents the magnetic polarization and the abscissa the magnetic field strength opposing the polarization. For a cylindrical magnet with height equal to diameter (*N*_m_ = 0.312 [9]), self-demagnetization shifts the operating point from the *J*-intercept (no opposing field strength) to the point indicated by a dot.

The supplier of our magnets provided a depolarization curve that shows how *J* is reduced in the presence of an opposing external field (Fig. 4). The shape of the magnet also has an effect on *J*. Even with no applied field, a cylindrical magnet will suffer to some extent from self-demagnetization if *γ* < ∞. In the case of a cylindrical magnet with *γ* = ∞ (height equal to diameter), the demagnetizing factor *N*_m_ is 0.312 [9]. Thus a first estimate of *m* is the volume of the magnet multiplied by the value of *M* corresponding to the dot in Fig. 4. Note that the demagnetizing factor of the magnet has a relatively small effect on *M*. This is an important property of rare-earth magnets.

Taking *M* and *V* from the manufacturer’s data sheets thus gives us the following estimate: *m* = 0.0898 A·m^2^. It is difficult to assign an uncertainty but we will see in Sec. 4.2.2 that experimental values are within 1.5 % of this.

The estimated value of *m* is useful in computing an approximate value for *H*_max_, the maximum field strength to which the sample will be exposed at a nominal setting of *Z*_0_. For 15 mm < *Z*_0_ < 30 mm, 4.25 kA·m^−1^ > *H*_max_ > 0.53 kA·m^−1^.

### 4.2 Bootstrap Calibration

**Fig. 5:**
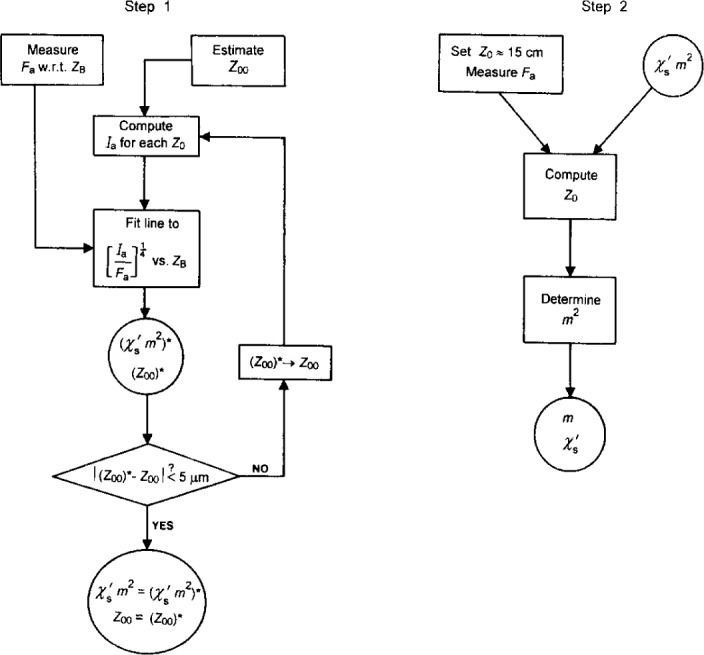
Flow charts showing the two steps of self-calibration. Step 1 determines the quantity *χ*′*_s_m*^2^ and Step 2 then determines the individual parameters *m* and *χ*′_s_. Computations shown in Step 1 can be greatly simplified through the use of commercial software.

The calculation of *m* given in Sec. 4.1 is a good approximation but the definitive value for *m* is best determined experimentally. In any case, we still require a routine method of finding *Z*_0_ to sufficient accuracy. Both needs can be met by carrying out an initial series of bootstrap measurements, shown schematically in Fig. 5 and described in detail below. We first determine *χ*′_s_*m*^2^ for a selected standard S. Based on Eqs. (4) and (5a), this quantity then allows us to find *Z*_0_ by placing the standard on the apparatus and measuring *F*_a_. Once we have a way of determining *Z*_0_, we can find *m*, as shown below. The bootstrap measurements require considerable effort but need only be done once. In the following, we analyze statistical uncertainties (i.e., Type A standard uncertainties) only. Type B standard uncertainties are discussed in Sec. 4.3.

#### 4.2.1 Determination of *χ'_s_m^2^* (and hence *Z*_0_)

Using gauge blocks (see Appendix A), *Z*_0_ can be incremented in precise steps of, for example, 5 mm. The value of *Z*_0_ may thus be known to within a constant length *Z*_00_ = *Z*_0_ − Z_B_, where *Z*_B_ is the height of the gauge blocks in use. If we had a suitable standard with known susceptibility *χ*_s_ and if, further, we knew *m*^2^, then a measurement of *F*_a_ using the standard would determine the value of *Z*_0_ each time the height of the stage was changed.

Standard samples of sufficiently large susceptibility are notoriously difficult to obtain [8] and so we standardize an unknown sample by measuring it at different settings of *Z*_0_ and fitting the results to the function predicted by the theory presented above. The parameters obtained from this fit contain some of the information we require. The force *F*_a_ computed from Eqs. (4), (5a) and (6a) depends on the dimensions of the standard as well as the quantities (*χ*′_s_*m*^2^) and *Z*_0_. For a suitable standard which is linear (up to field strengths of about 20 kA·m^−1^), isotropic and homogeneous, *F*_a_ as a function of *Z*_B_ may be fitted using two adjustable parameters and hence the unknowns *χ*′_s_*m*^2^ and *Z*_0_ can be found. (In the following we will use the abbreviation LIH to refer to materials that are magnetically linear, isotropic and homogeneous.) As long as we use the same magnet and the same sample, *χ*′_s_*m*^2^ will not, in the normal course of events, change with time and so the standard may be used to find *Z*_0_ whenever the height of the apparatus is changed.

Given a cylindrical sample that is LIH and has a susceptibility of about 0.0015, the major difficulties are:

- The computation of *I*_a_ requires a value for *Z*_00_, which is unknown. Thus all calculations must be carried out iteratively by making an initial guess of *Z*_00_.
- The functional form of *F*_a_ versus *Z*_B_ is not linear. A linear result will be obtained by plotting (*I*_a_/*F*_a_)^1/4^ versus *Z*_B_.
- As shown in Sec. 3.1, results obtained at relatively small values of *Z*_0_ are biased by assuming the cylindrical magnet to be a dipole.

The experimental algorithm is shown as Step 1 of Fig. 5. The sample we chose is made of the nonmagnetic alloy Alacrite X.S.H. (Aubert and Duval S. A., Neuilly sur Seine, France)^3^ whose nominal mass composition is: 20 % Cr, 15 % W, 10 % Ni, 0.1 % C, remainder Co. It is a polished disc with a diameter of 69 mm and a thickness of 9.8 mm and had already been fabricated for another purpose. In retrospect, a thickness of about 20 mm would have been preferable to increase the signal. Measurements of *F*_a_ were made starting at *Z*_0_ = *Z*_00_ ≈ 9.5 mm and in precise increments of 5 mm up to *Z*_0_ = Z_00_ + 20 mm.

Table 1 shows the final iterated values of *χ*′_s_*m*^2^ and *Z*_00_ based on three different least squares fitting routines. For fit (1), a weighted linear least-squares routine was used, where the uncertainty in *Z*_B_ is assumed to be negligible. Formally, we are fitting *y* = *β*_1_·*x* + *β*_0_ where $y={\left(\frac{I_{a}}{F_{a}}\right)}^{1/4}$ and *x* = *Z*_B_. Since *I*_a_ depends weakly on (*β*_0_/*β*_1_) + *x*, several iterations are required.

The datum at *Z*_0_ ≈ 9.5 mm, being the most biased by use of the dipole model and also being outside our normal range of use, was simply not used in the fit. Fit (2) differs from (1) in that equal weight was given to the four estimates of (*I*_a_/*F*_a_)^1/4^. Use was made of the covariance matrices for each fit in order to arrive at the tabulated standard uncertainties. We expect the equal weighting of fit (2) to yield unbiased estimates of the regression parameters but with a larger uncertainty compared to a weighted fit. The tabulated values confirm this expectation.

Fit (3) uses the curve-fitting utility of Sigmaplot 1.02 to fit the data directly to Eqs. (5a) and (6a). Formally, we are fitting $y=\frac{\lambda_{1}I_{a}}{{\left(\lambda_{0}+x\right)}^{4}}$ where *y* = *F*_a_, *x* = *Z*_B_, and *I*_a_ is an implicit function of *λ*_0_ + *x*. The results shown in Table 1 are for equal weight given to the data. Results obtained by weighting the input data are scarcely different. Again the point at *Z*_B_ = 0 has been omitted. The two parameters shown in the table are highly correlated in all three fits.

Taking fit (3) as our estimator, the final result is

$$\chi_{s}^{\prime }m^{2}=(10.70\pm 0.05)\times 10^{-6}A^{2}\cdot m^{4}.$$

We note that using the fitted values to extrapolate to *Z*_B_ = 0 results in a value of *F*_a_ that is about 5 % lower than the observed force. The implications of this error are discussed in Sec. 4.3.

#### 4.2.2 Determination of *m*

Given *χ*′_s_*m*, it is a relatively simple step to determine *m*, and hence *χ*′_s_ (Step 2 of Fig. 5). To do this, we need two additional magnets, B and C, of similar dimensions to A, the magnet we normally use. The additional magnets have moments that are, as yet, undetermined although all three moments are expected to be consistent with the estimate obtained in Sec. 4.1. We determine the magnetic moment of each magnet by using the susceptometer to measure the force between all possible pairs of magnets, aligned coaxially and placed a known distance apart.

We first set the span of the susceptometer so that *Z*_0_ is approximately 15 mm. A precise value of *Z*_0_ is determined from a measurement of *F*_a_ using the Alacrite standard whose calibration was described in Sec. 4.2.1. We then add gauge blocks with a height *Z*_B_ = 70 mm so that *Z*_0_ is nominally 85 mm.

At this setting, we measure the force between magnets A and B. Magnet B is placed on the span so that the resulting interaction force is attractive. Its orientation is then reversed so that the reaction force is repulsive. The same sequence of measurements is repeated for C. Ensuring that the two magnets are sufficiently coaxial is relatively straightforward because the coaxial condition gives a maximum change in the balance reading.

The average magnitudes of the attractive and repulsive forces give us *F*_b_ between magnets A, B and A, C. We then place magnet B on the pedestal and repeat the measurements by placing A and C on the span in each orientation to obtain *F*_b_ between B, C and B, A. The last measurement serves as a check on the reproducibility of the results. As may be inferred from Table 2, we found the balance readings were nominally 8 300 μg. The mean standard deviation of four repeated balance readings was 15 μg.

The force measured between magnets A and B is

$${\left(F_{b}\right)}_{\text{AB}}\equiv F_{\text{AB}}=\left(\frac{6\mu_{0}}{4\pi }\right)\frac{m_{A}m_{B}}{{\left(Z_{0}+L/2\right)}^{2}},$$

where *L*/2 is 2.5 mm, half the height of the magnet, and we have added subscripts to distinguish the moments of magnets A and B. The equation is the well known relation for the interaction force between two coaxial dipoles and is a special case of Eqs. (5a) and (6b) when *Z*_0_ is large compared with the sample dimensions and *χ* is negligible. Thus

$$m_{A}^{2}=\frac{F_{\text{AB}}\cdot F_{\text{AC}}}{F_{\text{BC}}}\cdot \frac{4\pi {\left(Z_{0}+L/2\right)}^{4}}{6\mu_{0}}.$$ (7)

The indices A, B, and C in Eq. (7) can be permuted in obvious fashion to obtain the moments of magnets B and C.

The results are given in Table 2. We note that, on average, the three magnetic moments agree well with the estimate of 0.0898 A·m^2^ obtained in Sec. 4.1.

With the value of *m* in hand, we can now compute *χ*′_s_ for the Alacrite standard. The result is

$$\chi_{s}^{\prime }=0.001\;348$$

with *u*c = 0.000 012 (Type B standard uncertainties neglected).

### 4.3 Calibration Checks

Once we have a standard with a known susceptibility, any height *Z*_0_ is determined by a measurement of *F*_a_(s), where (s) denotes use of the standard. The susceptibility *χ*′ of an object of interest is then determined through a measurement of the force *F*_a_ due to the unkown object placed at the same *Z*_0_. In essence, the following relation is used:

$$\frac{\chi^{\prime }}{\chi_{s}^{\prime }}=\frac{F_{a}}{F_{a}(s)}\cdot \frac{I_{a}(s)}{Ia}$$ (8)

where *I*_a_(s) is appropriate to the standard and *I*_a_ to the unknown [Eqs. (5b) and (6a)]. In Sec. 3.2 we referred to the special case where the standard and unknown have the same dimensions so that {*I*_a_(s)/*I*_a_} = 1 in general. This relation also holds for “large” samples as defined in Sec. 3.1. The ratio of susceptibilities is manifestly insensitive to details of the theory for such cases. It is only when the term {*I*_a_(s)/*I*_a_} depends sensitively on *Z*_0_ that we must rely on the quantitative validity of the theory developed above.

Two measurements described in detail in Ref. [1] provide checks on the method of calibration used in the previous section and help in the assessment of overall type B standard uncertainties. First, it was shown that the susceptibility measured for a number of well-characterized materials is generally in good agreement with handbook values. By itself, this is not a sufficient check of the apparatus because the susceptibilities of the standard materials used are relatively small in magnitude compared with the susceptibilities of stainless steels and other alloys of interest. Thus the standard materials can only be used to verify operation at relatively high fields, corresponding to values of *Z*_0_ in the range of 10 mm to 15 mm. In this range, the approximation of the magnet as a dipole is expected to bias results for samples that are not “large.” We expect good results for large samples, however, and this serves to check our inferred value of *χ*′_s_.

The second test is that the ratio of the measured susceptibilities of two different LIH alloys having significantly different dimensions and susceptibility is found to be independent of *Z*_0_ through a range of settings where {*I*_a_(s)/*I*_a_} changes by 50 %. These tests suggest that, for the examples given below, the type B relative standard uncertainty is no greater than 3 % in the measured susceptibility for LIH samples.

## 5. Typical Use of Susceptometer

We find it convenient to use the automatic-zero feature of the balance. The reading displayed is consequently insensitive to small, slowly changing forces. We therefore take the following precautions during the measurements. We do not collect data at settings where the introduction of the sample changes the balance reading by less than 10 counts in the last displayed digit. To change the position of the sample, we remove it entirely, let the balance return to zero and then place the sample in its new position. We repeat a measurement if the balance zero changes significantly during a sequence of readings.

For routine measurements, we begin with *Z*_0_ at no less than 25 mm and only decrease *Z*_0_ if the sample susceptibility is too small to produce a reasonable signal. Setting *Z*_0_ to 25 mm corresponds to a maximum field strength of *H*_max_ ≈ 900 A·m^−1^ (*μ* m*H*_max_ ≈ 1 mT).

Appendices B and C summarize susceptibility measurements for typical 1 kg mass standards of Class E_2_. The first (Appendix B) has the shape of a right-circular cylinder with rounded edges and the second (Appendix C) has the external dimensions of a Class M standard [6].

### 5.1 Cylindrical Mass Standard

In the example shown in Appendix B, the sample was placed coaxial with the magnet at a nominal spacing of 30 mm. Measurements were made using both vertical orientations of the magnet and both vertical orientations of the sample. Data obtained using the Alacrite standard were used to determine the average *Z*_0_, 30.55 mm. Equations (5a) and (6b) predict that the Alacrite standard should produce a value of *F*_b_/*g* = +5 μg. We observe +6 μg, which is a useful check that the device is operating properly.

As shown in Appendix B, the balance readings obtained when the bottom of the sample was nearer the magnet differ from those when the usual orientation of the sample is reversed. This could be a real difference due to sample inhomogeneity. However, the fact that *F*_a_ is essentially identical for each orientation of the sample suggests that permanent magnetization is the more probable explanation.

We emphasize that our calculation of *M_z_* assumes a model of uniform magnetization. Evidence that this model is unrealistic is that the fitted value of *M_z_* for the sample in its normal orientation is 0.2 A·m^−1^ but 0.07 A·m^−1^ when the sample is reversed.

Based on the limit derived in Sec. 2, we would not expect the observed magnetization to be of concern. To see if the sample magnetization has any practical consequences, we measured the mass of the secondary standard using a magnetically servocontrolled balance (Mettler-Toledo HK 1000 MC). The results obtained were the same when the standard was upside down on the balance. This means that, although the sample has a detectable permanent magnetization, routine weighings in our laboratory should be unaffected.

### 5.2 OIML-Shaped Mass Standard

Mass standards are not generally cylinders, although they do possess cylindrical symmetry. A typical shape is the so-called OIML design [6], shown in Appendix C. It differs from a simple cylinder primarily in that it has a lifting knob on top and a recessed base.

Appendix C presents calculations of *χ*′ for a 1 kg standard in its usual orientation carried out at a setting of *Z*_0_ typical for routine measurements. Both orientations of the magnet are used in order to obtain the value of *F*_a_. The same data are used to infer an apparent value of *M*_z_ from the calculation of *F*_b_, see Eq. (5).

To demonstrate the generality of the theory, we also made measurements with a similar mass standard placed on its side so that its body was centered directly over the magnet. Equation (5b) was solved numerically, with limits of integration corresponding to the cylindrical body of the standard. As shown in Table 6 (Appendix C), the presence of the knob has a relatively small effect on the calculation. Experimental results were unchanged when the sample was rotated about its axis of symmetry.

## 6. Behavior of Nonlinear and/or Inhomogeneous Samples

The previous section described typical measurements for alloys used in the manufacture of good secondary mass standards. We now give examples of results obtained from test objects that are nonlinear and/or inhomogeneous.

### 6.1 Nonlinear Alloy, Initially Unmagnetized

Pure brass is diamagnetic. We find that much of the industrial brass supplied to our workshops is, on the contrary, contaminated with magnetic impurities to the extent that the measured susceptibility is frequently greater than 0.01 in field strengths ranging up to about 1 kA·m^−1^. Typically, the susceptibility depnds on maximum field strength.

If one attempts to make the measurement at a maximum field strength of 10 kA·m^−1^ or more, the balance reading does not stabilize in the usual settling time but continues to increase in magnitude for many minutes. This is an indication that a portion of the sample is becoming permanently magnetized, as can be verified by carrying out further measurements with a maximum field an order of magnitude less strong. Exposure of poor-quality samples to high fields must, therefore, be avoided. This is the reason we begin all routine testing at low fields. For the same reason, we take great care that test objects do not accidentally come in contact with the permanent magnet.

### 6.2 Nonlinear Alloy, Initially Magnetized

It is not unusual to encounter samples of industrial-grade copper alloys or of type 304 stainless steel that have accidentally been magnetized at some point in their history. Once magnetized, stainless steels are difficult to demagnetize, as is well known [5, 10].

We found one disc of copper-beryllium alloy (mass fraction of beryllium nominally 2 %) that was magnetized to such an extent that the term in *M_z_* in Eq. (5a) dominated the measurements. Results for this sample are given in Table 3. It may be seen that the model of uniform magnetization fits reasonably well in this case, with *M_z_* about 25 A·m^−1^. Commercial alloys of Cu–2 %Be contain small but significant amounts of ferromagnetic elements. It is, therefore, the heat treatment given to the alloy that renders it more or less magnetic [11].

We then passed the sample through a demagnetizing coil energized at the main frequency (50 Hz). Results obtained after this operation are also given in Table 5. We see that, although still present, the permanent magnetization is weaker and evidently not as uniform. Data obtained at the two nearest settings of *Z*_0_ now reveal a measurable *F*_a_, indicating a susceptibility of order +0.000 35. This value is consistent with the range of susceptibilities common to commercial alloys of Cu–2 %Be [11].

### 6.3 Good-Quality Alloy, Surface Inhomogeneity

One of the well-characterized materials that we tested was a disc of oxygen-free copper with a mole fraction for iron impurity of only 2×10^−6^. This alloy has a handbook susceptibility of −9.6×10^−6^ [11]. The disc-shaped sample supplied by the manufacturer had rough-sawn faces that we machined flat before the measurements.

The first susceptometer reading appeared to show that the sample was paramagnetic. After it was given a light etch in a copper-cleaning solution, further measurements showed the sample to have the expected susceptibility. Thus the initial problem was a surface effect, presumably due to iron impurities transferred from the cutting tool. In order to have sufficient signal, these measurements were made at *Z*_0_ ≈ 10 mm.

Hard-working of stainless steel surfaces, as may happen during the fabrication of mass standards, can cause the susceptibility to increase [4]. Should this occur, the fabricated standard becomes inhomogeneous and use of Eq. (5b) will make it appear that the susceptibility of the sample is decreasing with increasing *Z*_0_. This may be the explanation of results reported in Ref. [1] for a cylinder of the stainless steel Immaculate V where the susceptibility inferred from Eq. (5) decreased by about 7 % as *Z*_0_ was increased from 9.3 mm to 24 mm. The susceptibility did not decrease significantly when *Z*_0_ was further increased to 29 mm. The sample had no detectable permanent magnetization.

We cannot take quantitative account of an enhanced surface susceptibility without prior knowledge of either the thickness of the enhanced layer or its susceptibility.

## 7. Magnetic Fields in a Balance

It is possible to measure both the vertical magnetic field intensity and its gradient in the weighing chamber of the MT5 balance used for these measurements. Using a conventional magnetometer, we find the vertical field within the weighing chamber (with the cylindrical magnet removed) is about 10 % greater than that of the Earth alone.

The gradient of this field, measured by raising the probe a few centimeters, was inferred to be of order 40 A·m^−2^. An independent measure of the gradient is obtained by observing the change in balance reading when the magnet is reversed with no sample present. The measured difference is 1.5 mg implying a gradient of 65 A·m^−2^ (note that the difference in balance reading upon reversal of the magnet reported in Ref. [1] was 0.9 mg; this was obtained from a different unit of the same model balance).

Thus, based on Eqs. (2) and (3), if the balance is used to weigh 5 g of stainless steel, the susceptibility of the material should be less than 5 and any uniform magnetization in the vertical direction should be less than 200 A·m^−1^ in order to keep magnetic effects below the balance resolution of 1 mg. If the same average field strength and gradient were present in a 1 kg mass comparator, stainless steel standards would require *χ* < 0.025 and |*M_z_*| < 1 A·m^−1^ in order to keep magnetic effects below 1 μg.

## 8. Conclusion

We have discussed how magnetic forces may lead to errors in mass measurements. The force equations that describe the unwanted effects also describe the operation of the BIPM susceptometer. We have presented design details of this device, which is suitable for checking the susceptibility of 1 kg mass standards of stainless steel and similar nonmagnetic or weakly magnetic alloys.

In addition, we have demonstrated that the device may be calibrated by using a set of gauge blocks, two additional magnets, and a LIH sample of unknown susceptibility. The calibration was verified by measurements, in relatively high field strengths, of materials having well-characterized susceptibilities.

The device can also detect a permanent magnetization. An apparent magnetization can be calculated and has a simple interpretation for uniformly magnetized samples. An acceptable upper limit for uniform magnetization has been derived in the spirit of a previous discussion of an acceptable upper limit for the susceptibility.

## Figures and Tables

**Fig. 6:**
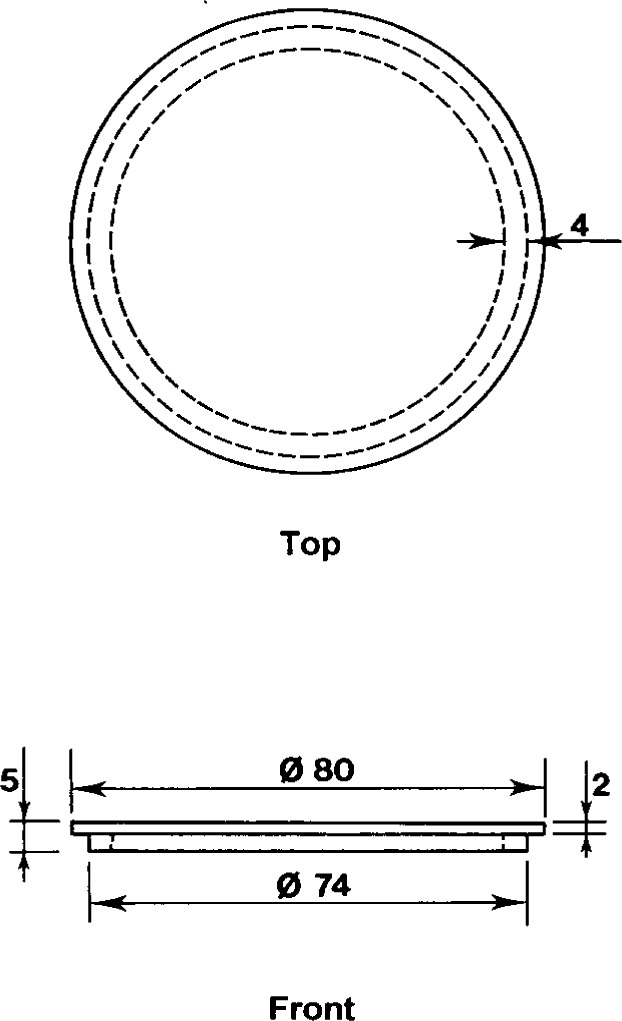
Replacement cover for the balance, made by gluing an annular piece of aluminum alloy (AU4G) to the bottom of a thin disc of the same material. Dimensions are in millimeters.

**Fig. 7:**
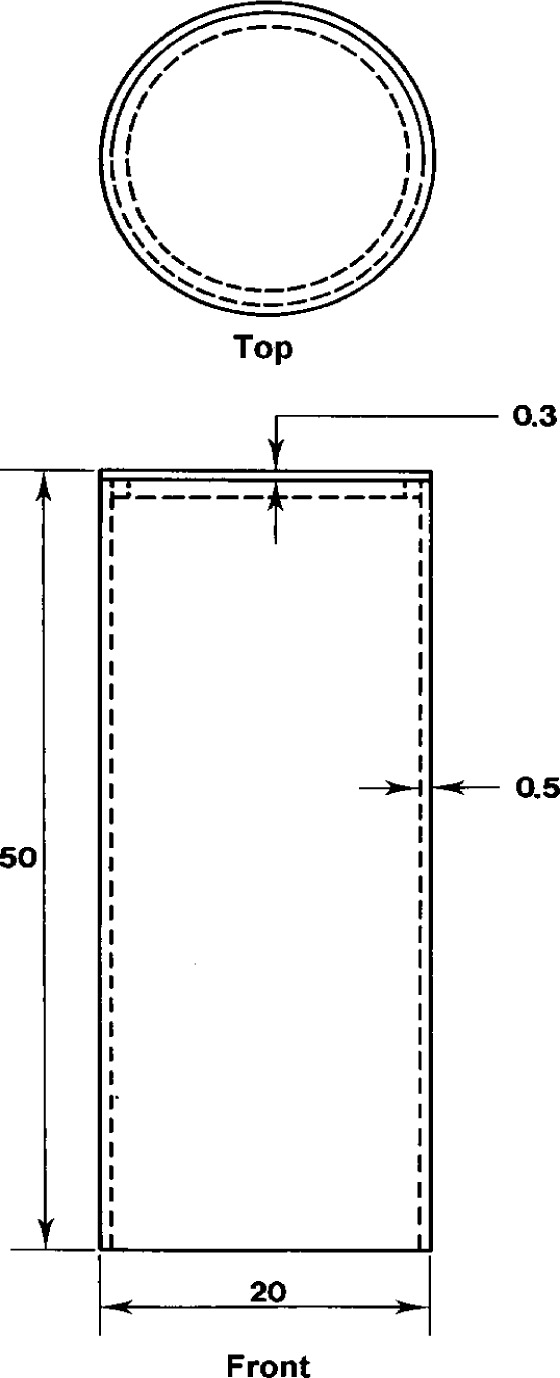
Pedestal used to support the magnet. Not shown are 48 holes (eight columns of six), 5 mm in diameter, that are drilled into the tube in order to reduce its mass. The material used for the pedestal is AU4G; dimensions are in millimeters.

**Fig. 8:**
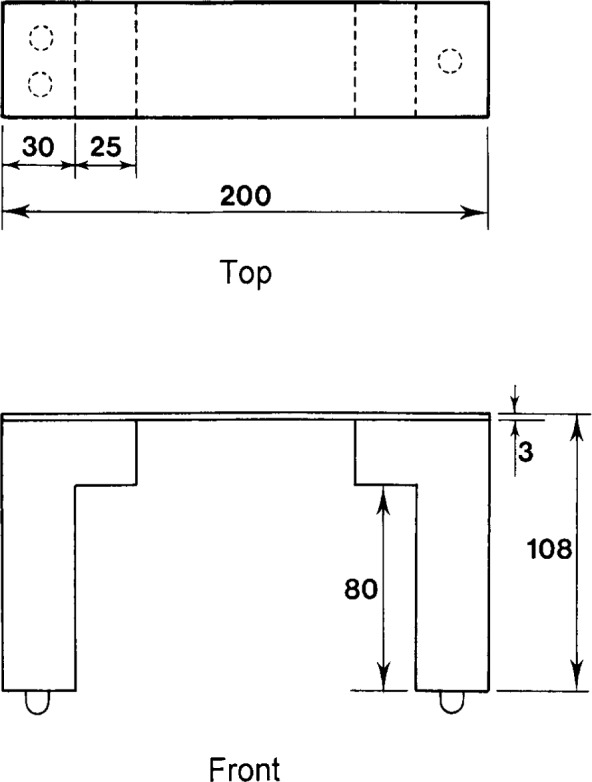
Bridge used to support the test samples. Not shown are screws that are used to fasten the horizontal span to the two vertical supports. Also not shown are fiducial marks lightly inscribed on the top surface of the span and used as an aid in centering samples. The material used is AU4G except for the screws, which are brass. Dimensions are in millimeters.

**Fig. 9:**
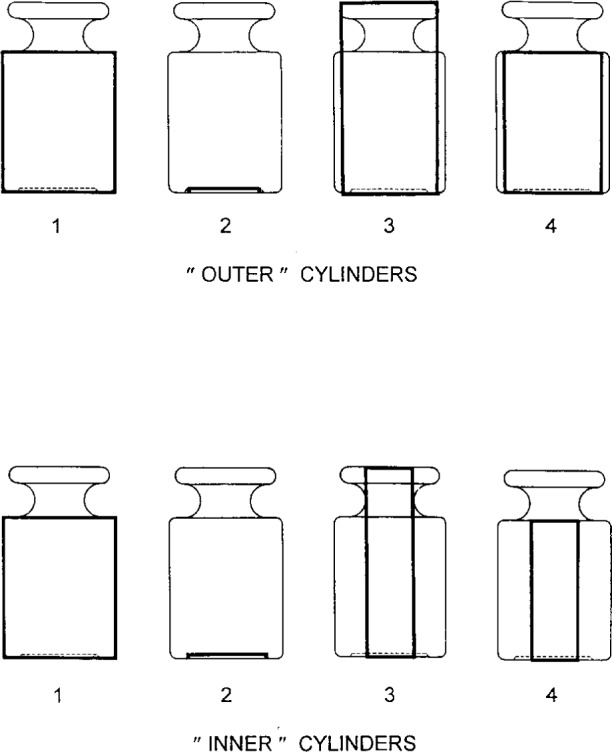
Outer and inner cylinders used to approximate an OIML-shaped 1 kg mass standard by the method of superposition. Dimensions of the cylinders are given in Table 5.

**Table 1:** Data and data analyses for determination of the quantity $\chi_{s}^{\prime }m^{2}$ from measurements of *F*_a_ as a function of *Z*_B_. Standard uncertainties for the measurements of *F*_a_ are given in the second column

*F*_a_/*μ*N^a^	*u*_c_/*μ*N	*Z*_B_/mm
24.65	0.07	0
3.990	0.0012	5
1.112	0.010	10
0.4042	0.005	15
0.1702	0.005	20

Fit number^b^			
	1	2	3
*χ*′_s_*m*^2^/(A^2^m^4^)	10.718×10^−6^	10.539×10^−6^	10.703×10^−6^
*σ*/(A^2^m^4^)	0.069×10^−6^	0.10×10^−6^	0.044×10^−6^
*Z*_00_/mm	9.477	9.404	9.470
*σ*/mm	0.024	0.075	0.014
Deg. freedom	2	2	2

a +1 μN corresponds to a balance reading of −101.9 μg.

b The three fits shown in the table result from different ways of manipulating the same data. Fit (3), which was carried out using commercial software, is taken as the definitive result.

**Table 2:** Calculation of magnetic moments *m* of magnets A, B and C based on the force *F*_b_ measured between pairs of magnets aligned at a spacing of 84.90 mm

Magnets	*F*_b_/μN^a^ (measured)	*m*/(A·m^2^) (calculated)
A, B	81.80	
A, C	83.35	
B, C	83.50	
A, B	81.86	
A		0.08910
B		0.08937
C		0.09089

a +1 μN corresponds to a balance reading of −101.9 μg.

**Table 3:** Effect of “degaussing” on a magnetized disc of commercial-grade Cu–2 %Be. The influence of the Earth’s field on the calculation of *M_z_* may be neglected

Before degaussing			After degaussing			

Nominal *Z*_0_/mm	Measured *F*_b_/μN^a^	*M_z_*/(A·m^−1^)	Measured *F*_a_/μN	*χ*/10^−4^	Measured *F*_b_/μN	*M_z_*/(A·m^−1^)
15			0.96	3.52	7.51	13.2
20	12.68	25.5	0.27	3.40	5.80	12.1
25	10.06	25.2				
30	7.72	24.9			3.14	10.7
40	4.395	24.4				
50	2.531	24.0			0.97	9.7
70	0.963	24.0			0.37	9.7
90	0.404	22.8				

a +1 μN corresponds to a balance reading of −101.9 μg.

**Table 4:** Computation of *χ* and *M_z_* for a 1 kg stainless steel standard of cylindrical shape. The height and diameter of the cylinder are both 54 mm and the edges are chamfered with a radius of 2 mm. Measurements were made with the cylinder coaxial to the magnet at a spacing of *Z*_0_ = 30.57 mm

Experimental data			

Sample orientation	*F*_a_/μN^a^		*F*_b_/μN
Normal	0.902		0.489
Reversed	0.904		0.199

Calculation of *χ*			

Sample dimensions	*I*_a_		*χ*

Height = diameter = 54 mm	0.773		0.006 85
Height = 54 mm; diameter = 50 mm	0.733		0.007 21
Height = diameter = 54 mm	0.773		
Height = 2 mm; diameter = 54 mm	−0.199		
Height = 2 mm; diameter = 50 mm	+0.192		
Correction for lower edge	−0.007 (1–*π*/4) = −0.002		
Total	0.771		0.006 86

Calculation of *M_z_*			

Sample orientation	Measured *F*_b_/μN	Theoretical effect of $H_{e_{z}}/\mu N$	Adjusted *M_z_*/(A · m^−1^)

Normal	0.489	0.275	0.20
Reversed	0.199	0.275	0.07

a +1 μN corresponds to a balance reading of −101.9 μg.

**Table 5:** Computation of *χ* and *M_x_* for an OIML-shaped 1 kg stainless steel standard. Details of the shape are given in Fig. 9. Measurements were made with the cylinder coaxial to the magnet at a spacing of *Z*_0_ = 24.70 mm

Measured values *F*_a_ = 1.034 μN^a^ *F*_b_ = 0.169 μN				

Calculation of *χ*				

	*c/*mm	*t*/mm	*I*_a_	*χ*
Outer cylinders				
1	24.0	58.5	0.8196	
2	16.5	1.0	0.1116	
3	21.5	80.5	0.7690	
4	21.5	58.5	−0.7681	
		Total	0.7089	0.00364

Inner cylinders				
1	24.0	58.5	0.8196	
2	17.5	1.0	−0.1164	
3	13.5	80.5	0.4979	
4	13.5	58.5	−0.4975	
		Total	0.7036	0.00367

Calculation of *M_z_*				

Sample dimensions		Measured *F*_b_/μN	Theoretical effect of $H_{e_{z}}/\mu N$	Adjusted *M_z_*/(A · m^−1^)

OIML				
Cylinder, diam. = 48 mm; ht. = 59 mm correction for cylindrical recess			0.199	
			−0.012	
Correction for bevel			−0.001	
	Total	0.169	0.186	≤ 0.07

a +1 μN corresponds to a balance reading of −101.9 μg.

**Table 6:** Calculations for horizontal orientations of the mass standard

c/mm	*t*/mm	*I*_a_	*χ*
24	59	0.661	0.003 68
24	21 + 59 + 21	0.684	0.003 55
24	21 + 59	0.672	0.003 62

## Footnotes

1 This assumes that the laboratory is not magnetically shielded and that no extraneous fields are present. These assumptions were verified for our laboratory.

2 Equation (8) of [1] has obvious typesetting errors.

3 Certain commercial equipment, instruments, or materials are identified in this paper to foster understanding. Such identification does not imply recommendation or endorsement by the National Institute of Standards and Technology, nor does it imply that the materials or equipment identified are necessarily the best available for the purpose.

## 9. Appendix A. Construction of the Apparatus

The apparatus is shown schematically in Fig. 1 and the most important construction details are given below.

### 9.1 Balance

The balance used (Mettler-Toledo AG, model MT5) has a 5 g capacity and a resolution of 1 μg. The weighing chamber is a glass tube with a removable glass top. We replace the original top with one made of an aluminum alloy, as shown in Fig. 6. The alloy we use, designated AU4G in France, corresponds closely to ASTM 2017 and is the aluminum alloy commonly stocked by our workshop.

### 9.2 Magnet

The cylindrical magnets used are made of neodymium-iron-boron (Vacuumschmelz GmbH, type 370 HR). The dimensions of each magnet are height = 2.5 mm; diameter = 5 mm. The axis of magnetization coincides with the geometric axis of the cylinder. The density of the NdFeB alloy is 7400 kg·m^−3^. Two such magnets are combined to produce a cylinder of height equal to diameter. In all equations given in the text, the origin of the coordinate system is the geometric center of the magnet.

When in doubt, the north pole of the magnet can easily be located by placing the magnet horizontally on a smooth, nonmagnetic surface. It then acts like a compass, spontaneously aligning itself along the Earth’s horizontal field.

### 9.3 Pedestal

The magnet sits on a tubular pedestal of AU4G that is itself centered on the balance pan. Pedestal dimensions are shown in Fig. 7. Holes (not shown) drilled through the wall of the tube ensure that the total mass of the magnet and pedestal is well within the capacity of the balance.

An aluminum shim can be used to raise the magnet closer to the balance cover so that *Z*_0_ is reduced to a minimum, thereby achieving maximum sensitivity. The shim is not used in routine work. The total mass of the magnets, pedestal and shim is about 4.2 g.

### 9.4 Bridge

Samples are centered on a bridge, also made of AU4G, that straddles the weighing chamber of the balance. Details are shown in Fig. 8. The important features of the design are that the span can be made level with respect to the balance pan and that the thickness of the span is the minimum consistent with adequate mechanical rigidity. If the span sags in the middle, samples of large diameter will be farther from the magnet than samples of small diameter. For critical measurements, a diamagnetic shim of small diameter can be placed at the center of the span thereby overcoming the problem of sag.

### 9.5 Gauge Blocks

We have found it convenient to place the bridge on nonmagnetic gauge blocks so that it may be raised or lowered in precise increments of 5 mm. We manufacture our blocks either of AU4G or of selected brass. The selection procedure consists of placing the brass stock in the vicinity where the eventual gauge block will be placed and verifying that there is no change in the balance reading with the magnet installed.

### 9.6 UMT5 Balance

A limited set of measurements was made using a model UMT5 balance (Mettler-Toledo AG), which has the same capacity and overall dimensions as the MT5 but has a resolution of 0.1 μg. The pan supplied with the UMT5 has a diameter of only 16 mm and, in order to use the pedestal shown in Fig. 7, the original pan was replaced by the pan from an MT5. Results obtained were satisfactory and indicate that the increased resolution allows the determination of the susceptibilities of samples of correspondingly smaller volumes.

## 10. Appendix B. Typical Calculations for a Cylindrical Mass Standard Coaxial with the Magnet

The following describes how we routinely determine the susceptibility and effective permanent magnetization of a cylinder oriented coaxially above the magnet (Fig. 1). We find it convenient to work with spreadsheet software that calculates: *F*_a_, from Eqs. (4), (5) and (6) as well as *F*_b_, from Eqs. (5) and (6). The value of *F*_b_ is displayed as two separate components: from the Earth’s vertical magnetic field *H*_E_*_z_* and a possible permanent magnetization *M_z_* coaxial with the sample. As we discuss below and in Sec. 11, it is also useful to display *I*_a_ calculated from Eq. (5a). This is a number between 1 and 0 that takes account of the finite dimensions of the sample relative to a semi-infinite slab. Spreadsheet variables are the radius *c* and length *t* of the cylindrical sample, *Z*_0_, *χ*, and *M_z_*.

To find *Z*_0_, we start with the measurement of *F*_a_ made with the Alacrite standard. With *c*, *t* and *χ*′_s_ appropriate to this sample, we call upon a spreadsheet utility to compute the value of *Z*_0_, through iterations of Eqs. (5a) and (6a), forcing the calculated value of *F*_a_ to agree with that found by experiment. We check that *F*_b_ with *M_z_* = 0 is consistent with the computed result.

We then enter *c* and *t* appropriate to the sample under test and, calling once more on the spreadsheet utility, adjust *χ* until the measured value of *F*_a_ is obtained. If necessary, we also adjust the value of *M_z_* until the calculated force *F*_b_ agrees with experiment. A separate calculation for *x* and *M_z_* is carried out for the sample in its normal orientation and turned upside down.

Typically, the sample under test has edges that are rounded with a radius of about 2 mm. To check whether the actual shape produces the same signal as a cylinder with sharp edges, we make supplementary calculations shown in Table 4.

The simplest calculation, which is usually sufficient, is to set limits on the susceptibility. That is, the susceptibility must be less than that computed assuming a perfect cylinder of radius *c* and length *t*. The susceptibility must be greater than that computed for a perfect cylinder of radius (*c–*2 mm) and length *t*. The results of these calculations are shown in Table 4. It is sufficient to calculate *I*_a_ since *χ* is then found directly from Eq. (8).

We can also calculate the susceptibility more precisely. By superposition, we first determine *I*_a_ due to the smallest annular volume that contains the lower rounded edge. The contribution is, in fact, small compared with the total *I*_a_. We may be satisfied with this approximation; or we may note that all parts of the annulus are approximately equidistant from the magnet so that the signal from any arbitrarily chosen region of the annulus is roughly proportional to the volume of the region, as may be inferred from Eq. (5b). In particular, the material cut away from the annulus to make the rounded edge has a volume that is (1–*π*/4) times the total annular volume. Thus we have underestimated *χ* by about 0.1 %, an error that is well below the uncertainty of the final result. The correction for the upper rounded edge is even smaller, so we neglect it.

## 11. Appendix C. Typical Calculations for an OIML-Shaped Mass Standard

The mass standard is shown in Fig. 9. The lifting knob and the recessed base are complications that are dealt with in detail in this section. Two orientations of the sample are now considered.

### 11.1 Sample Coaxial With the Magnet

The usual experimental orientation we use when determining magnetic susceptibility is coaxial with the magnet. We first measure *Z*_0_ as described in Appendix B. It is then possible to put simple limits on the susceptibility: it must be less than that based on a sum and difference of “outer” cylinders that contain the entire volume of the standard; it must be greater than that based on a sum and difference of “inner” cylinders that are contained within the mass standard (see Fig. 9). The results are given in Table 5. Note that the net contribution of the knob is virtually negligible.

The same value of *Z*_0_ was used in calculating each component of *I*_a_. In general, any component *I*′_a_ ≡ *I*_a_(*Z′*_0_) calculated using a different distance *Z′*_0_ must be normalized by a factor (*Z*_0_/*Z′*_0_)^4^ before combining it with other components calculated using *Z*_0_. The reason for this is easy to see: Consider an example where there are only two components in the summation, *I*_a_ and *I′*_a_, computed at distances *Z*_0_ and *Z′*_0_ respectively. From Eq. (5a) and the principle of superposition, the total measured force must be described by the relation

$$F_{\text{total}}=[\chi^{\prime }F_{\max}(Z_{0})]\cdot I_{a}+[\chi^{\prime }F_{\max}(Z_{0}^{\prime })]\cdot I_{a}^{\prime }.$$ (9)

By simple algebra,

$$F_{\text{total}}=[\chi^{\prime }F_{\max}(Z_{0})]\cdot \left[I_{a}+{\left(\frac{Z_{0}^{\prime }}{Z_{0}}\right)}^{4}I_{a}^{\prime }\right].$$ (10)

Equation (10) can easily be generalized to an arbitrary number of components.

Once the total *I*_a_ has been found, we can determine *x* from Eqs. (4) and (5a). Since *F*_b_ is a relatively small number, we may approximate the sample as a cylinder resting on an annulus for this calculation. A more detailed calculation, shown in Table 5, verifies this assertion.

### 11.2 Axis of Sample Perpendicular to Axis of Magnet

It is possible to measure the susceptibility of a sample laid on its side, its body centered about the magnet axis. Our approach is, again, to calculate *I*_a_ and then solve for *χ* using Eqs. (4) and (5a). In this case, however, *I*_a_ is determined from (5b) by numerical differentiation of the volume integral, after first converting to Cartesian coordinates. The computation can be done using packaged software such as Mathcad:

$$\begin{array}{l} Z_{0n}=1\\ e=10^{-8}\\ I_{a}=-\frac{8}{3\pi }[\frac{\partial }{\partial Z_{0n}}\int_{Z_{0n}}^{Z_{0n}+2c_{n}}\int_{0}^{\sqrt{c_{n}^{2}-{\left(Z_{0n}+c_{n}-z\right)}^{2}+e}}\\ \;\int_{0}^{\frac{t_{n}}{2}}\frac{4z^{2}+x^{2}+y^{2}}{{\left(x^{2}+y^{2}+z^{2}\right)}^{4}}dxdydz]. \end{array}$$

All integration limits are dimensionless, having first been normalized to *Z*_0_ = 25.39 mm. The small quantity *e* is added to ensure that the upper limit of the second integral is real. Integration is over the dummy variables *x*, *y* and *z* representing the two horizontal axes and the vertical axis of a cartesian coordinate system where the *x*-axis is parallel to the sample axis. Computing time can be shortened considerably by analytical integration over the *x*-variable, a result that is found in standard references on integral calculus. The results of the calculation are shown in Table 6. The measured value of the attractive force was *F*_a_ = 0.871 μN. The first calculation excludes the knob in the calculation of *I*_a_ and therefore overestimates *χ*. The second assumes there is a knob on each end of the body of the standard and that each knob is a simple extension of the body by 20 mm; this underestimates *χ*. The final calculation (the average of the previous two) assumes a single knob but models it again as a simple extension of the body. This again overestimates *χ* but gives a tighter bound. Therefore 0.003 68 > *χ* > 0.003 62.

If the sample were permanently magnetized perpendicular to its axis of symmetry, the measurements of *F*_b_ would depend on the azimuth of the sample and this can easily be checked. It is, of course, possible to estimate by numerical methods the expected value of *F*_b_ due simply to the Earth’s field.

The measurements summarized in Tables 5 and 6 were made on different 1 kg standards fabricated from the same alloy.
