# Erratum: Determining the Magnetic Properties of 1 kg Mass Standards

**Published:** 1995

**Authors:** Richard S. Davis

**Affiliations:** Bureau International des Poids et Mesures, Pavillon de Breteuil, F-92312 Sèvres Cedex, France

[J. Res. Natl. Inst. Stand. Technol. Volume 100, Number 3, May–June 1995, p. 209]

Page 211, above Eq. (3).

Replace “the magnetic field within the balance chamber is vertical with the form…” by “the vertical magnetic field strength within the balance chamber has the form…”.

Page 217, Eq. (8).

Replace “*I*a” by “*I*_a_”.

Page 218, two paragraphs above Sec. 5.1. Replace “*μ*m*H*_max_” by “*μ*_0_*H*_max_”.

Page 223, first paragraph of Sec. 11.1.

Replace “: it must be less than that based on a sum…; it must be greater than that based on a sum…” by “: it must be greater than that based on a sum…; it must be less than that based on a sum…”.

Page 224, Table 5, column 4, row 2.

Replace “0.1116” by “−0.1116”.

Page 224, Eq. (10) should be:

$$F_{\text{total}}=\left[\chi^{\prime }F_{\max}\left(Z_{0}\right)\right]\cdot \left[I_{a}+{\left(\frac{Z_{0}}{Z_{0}^{\prime }}\right)}^{4}I_{a}^{\prime }\right].$$

Page 225, two paragraphs above the Acknowledgment. Replace “This again overestimates *χ*” by “This again underestimates *χ*”.

